# Local adaptation and rapid evolution of aphids in response to genetic interactions with their cottonwood hosts

**DOI:** 10.1002/ece3.6709

**Published:** 2020-09-02

**Authors:** Stuart C. Wooley, David Solance Smith, Eric V. Lonsdorf, Sarah C. Brown, Thomas G. Whitham, Stephen M. Shuster, Richard L. Lindroth

**Affiliations:** ^1^ Department of Entomology University of Wisconsin‐Madison Madison Wisconsin USA; ^2^ Department of Biological Sciences California State University Turlock California USA; ^3^ Department of Biological Sciences Northern Arizona University Flagstaff Arizona USA; ^4^ Biology Department California State University San Bernardino San Bernardino California USA; ^5^ Alexander Center for Population Biology Conservation and Science Lincoln Park Zoo Chicago Illinois USA; ^6^ Urban Wildlife Institute Conservation and Science Lincoln Park Zoo Chicago Illinois USA; ^7^ Center for Adaptable Western Landscapes Northern Arizona University Flagstaff Arizona USA

**Keywords:** aphid, arthropod, community genetics, cottonwood, geographic mosaic, insect, local adaptation, *Populus*, rapid evolution, selection mosaic

## Abstract

Several studies have demonstrated the ecological consequences of genetic variation within a single plant species. For example, these studies show that individual plant genotypes support unique composition of the plants' associated arthropod community. By contrast, fewer studies have explored how plant genetic variation may influence evolutionary dynamics in the plant's associated species. Here, we examine how aphids respond evolutionarily to genetic variation in their host plant. We conducted two experiments to examine local adaptation and rapid evolution of the free‐feeding aphid *Chaitophorus populicola* across genetic variants of its host plant, *Populus angustifolia*. To test for local adaptation, we collected tree cuttings and aphid colonies from three sites along an elevation/climate gradient and conducted a reciprocal transplant experiment. In general, home aphids (aphids transplanted onto trees from the same site) produced 1.7–3.4 times as many offspring as foreign aphids (aphids transplanted onto trees from different sites). To test for rapid evolution, we used 4 clonally replicated aphid genotypes and transplanted each onto 5 clonally replicated *P. angustifolia* genotypes. Each tree genotype started with the same aphid genotype composition. After 21 days (~two aphid generations), aphid genotype composition changed (i.e., aphids evolved) and some tree genotypes supported unique evolutionary trajectories of aphids. These results suggest that plant evolution in response to human perturbation, such as climate change and invasive species, will also result in evolutionary responses in strongly interacting species that could cascade to affect whole communities.

## Graphical abstract

We conducted two experiments to examine local adaptation and rapid evolution of the free‐feeding aphid *Chaitophorus populicola* across a genetic gradient of its host plant, *Populus angustifolia*. We found evidence of local adaptation, such that local aphids produced 1.7–3.4 times as many offspring as foreign aphids. Further, in just 21 days aphids rapidly and differentially evolved on different genotypes of their host plant.

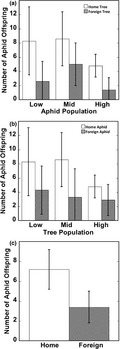

## 1 INTRODUCTION

Plant genetic variation plays a major role in influencing herbivore population dynamics and the structure of dependent communities (e.g., Johnson, 2008; Keith, Bailey, & Whitham, 2010; Maddox & Root, 1987; Smith, Bailey, Shuster, & Whitham, 2011; Smith et al., 2015). For example, plant genotypes differ in their quality as aphid hosts (Bailey, Wooley, Lindroth, & Whitham, 2006; Figueroa et al., 2004; Service, 1984; Smith et al., 2011; Whitham, 1989), which can have cascading effects to influence a much larger community (Keith, Bailey, Lau, & Whitham, 2017). Several studies have addressed the role of plant genetic variation in shaping herbivore dynamics. These studies have been performed in a variety of contexts, including agricultural systems (Figueroa et al., 2004; Jack & Friesen, 2019; Via, 1990; Via & Shaw, 1996; Vorburger, 2006; Vorburger, Lancaster, & Sunnucks, 2003), natural systems (Turcotte, Reznick, & Hare, 2011), and common garden (Karban, 1989; Moran, 1981; Pilson & Rausher, 1995; Turley & Johnson, 2015) and greenhouse settings (Laukkanen et al., 2012).

Conversely, arthropod genotypes can vary in their ability to live on different host‐plant species (Via & Hawthorne, 2002; Vorburger, 2006) and genotypes of the same plant species (Evans, Allan, Shuster, Woolbright, & Whitham, 2008; Figueroa et al., 2004; Garrido, Andraca‐Gómez, & Fornoni, 2012; McIntyre & Whitham, 2003). For example, Blackman (1990) reviewed 41 studies of aphid genotype and plant variety or species interactions. Of those, 56% showed significant evidence of specific associations between particular aphid genotypes and species or varieties of host plants. More recent studies have shown that selection can have strong effects on the genotypic frequencies of herbivore populations (Jin et al., 2015; Tanaka, Murata, & Matsuura, 2015; Turcotte et al., 2011; Vorburger, 2006). Such changes in gene or genotype frequencies within a season or a few generations would be considered rapid evolution (Schoener, 2011; Tanaka et al., 2015; Thompson, 1998; Turcotte et al., 2011).

Further, differential reproduction (i.e., natural selection) and evolution of herbivores could lead to local adaptation of herbivores to particular individuals, genotypes, or populations of their host plants. Those studies that have looked have found mixed evidence of herbivore adaptation to their host plant (Evans et al., 2008; Garrido et al., 2012; Karban, 1989; Laukkanen, Kalske, Muola, Leimu, & Mutikainen, 2018; Laukkanen et al., 2012; Mopper, 1996; Strauss, 1997). For example, Garrido et al. (2012) examined the relationship of four populations of an herbivore and its host plant. In two populations, they found evidence that the herbivores were adapted to the local plant population, but in one population, the herbivore was maladapted to the local host‐plant population, and in the fourth population, they found no evidence for adaptation (Garrido et al., 2012) suggesting a geographic mosaic of evolutionary responses of herbivores to their host plant (Thompson, 2005). These mixed results highlight the complexities of the process of adaptive evolution and the need to study conditions that promote and inhibit local adaptation.

**FIGURE 1:**
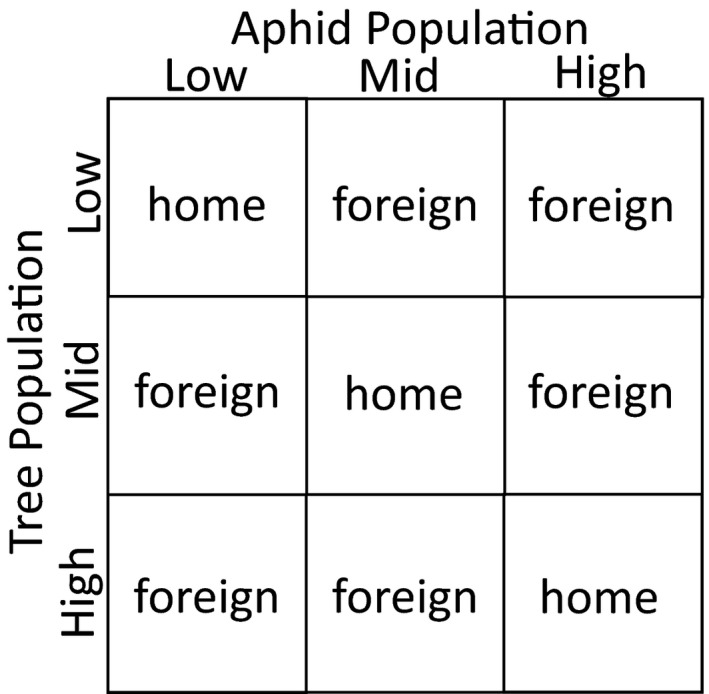
Schematic showing the experimental design for the reciprocal transplant experiment used to evaluate hypotheses of local adaptation. The label at the top designates the population (elevation) of the source of the aphids. The label along the left side designates the population (elevation) of the source of the trees. The word in each of the nine cells indicates the aphid–tree combination such that “home” describes aphids placed on trees from the same source population (elevation) and “foreign” describes aphids placed on trees from different source populations (elevations)

Kawecki and Ebert (2004) and Blanquart, Kaltz, Nuismer, and Gandon (2013) proposed three patterns that provide evidence of local adaptation when using a reciprocal transplant experiment. We describe these in the context of aphid populations being locally adapted to their host‐tree populations. First, if local adaptation has occurred, using pairs of tree populations, we expected aphids to produce more offspring when transplanted onto home tree populations compared with when they were transplanted onto foreign tree populations (Figure 1, comparing “home” aphids to “foreign” aphids in the same columns). This first pattern emphasizes variation in trees as aphid hosts and suggests that aphids have adapted to particular traits in their host‐plant population. Second, if local adaptation has occurred, we expected home aphids to produce more offspring than foreign aphids when both are placed on the same tree population (i.e., within tree parings of local and foreign aphid populations; Figure 1, comparing “home” to “foreign” in the same rows). This perspective emphasizes variation in aphids and shows that local aphids reproduced more than foreign aphids. Kawecki and Ebert (2004) argued that this second pattern is the strongest evidence for local adaptation as it shows the product of natural selection (differential fitness among aphid genotypes) within a single habitat (in this case, tree population). Third, if aphids are locally adapted, we expected the average reproduction of all home (i.e., sympatric) aphids to be higher than the average of all foreign (i.e., allopatric) aphids (Figure 1, comparing the main home diagonal to foreign off diagonal; Smith et al., 2012; Blanquart et al., 2013). This perspective is different from the previous two because, it averages across the effect of individual populations of aphids and trees to look at the general (average) trend of reproduction of local and foreign aphids. Blanquart et al. (2013) argued that this last pattern is the best evidence for local adaptation as it controls for the effects of aphid and tree population that can influence aphid fitness, but not necessarily in an adaptive way. One caveat of this last approach is that averaging across multiple populations assumes that plant–aphid interactions are relatively similar across sites. Previous studies have shown that species interactions and patterns of selection often vary across space (Smith et al., 2011; Thompson, 2005). Because averaging can cover important variation, results from the last approach should be interpreted accordingly. Importantly, any one of these three patterns would provide evidence of local adaptation. In this study, we examine all three patterns.

The use of multiple methods to detect local adaptation may produce inconsistent results; one method may provide evidence for local adaptation at both individual population and individual genotype levels, but another may not. This problem could arise because of a type II error, where one method may not detect local adaptation, even though it exists. Possible conflicting results like these, as well as advantages and disadvantages of the three methods used to detect local adaptation, are described in more detail in Kawecki and Ebert (2004) and Blanquart et al. (2013). The potential for inconsistent results emphasizes the need to use multiple approaches to investigate local adaptation. Doing so will provide a clearer picture of the effects of aphid population and tree population in influencing variation in fitness and could reveal instances where some, but not all populations are locally adapted (e.g., Garrido et al., 2012).

### 1.1 Hypotheses

We conducted two independent but related experiments at both individual tree population and individual tree genotype levels. The first experiment was designed to test the hypothesis that aphids are adapted to their local (home) host‐tree populations. Working with three populations of the host tree, *Populus angustifolia*, that are known to vary genetically along an elevation/climatic gradient (Evans et al., 2016; Martinsen, Whitham, Turek, & Keim, 2001), we conducted a reciprocal transplant experiment in a greenhouse, using aphid populations from the same three populations of trees. The second experiment, in an outdoor common garden, was designed to test the hypothesis that aphid evolution could rapidly and differentially occur among genotypes of the host tree. Findings of a change in the relative frequency of aphid genotypes in a single season would constitute evidence of rapid evolution (Thompson, 1998; Turcotte et al., 2011; Turcotte, Reznick, & Hare, 2013). Confirmation from both experiments is important as they address both the potential of rapid evolution and a geographic mosaic of evolution in which different aphid populations have different evolutionary trajectories on their host populations across the landscape (Thompson, 2005) and at a finer scale among individual genotypes.

## 2 M**ATERIALS AND METHODS**

### 2.1 Study system

Cottonwood trees (*Populus* spp.) are “foundation species,” that is, they structure their associated communities by creating locally stable conditions for other species, and by modulating and stabilizing fundamental ecosystem processes (Dayton, 1972; Ellison et al., 2005). Previous studies have measured significant genetic variation within and among *Populus* populations. Some of this variation may be the result of climate‐driven divergent selection and adaptive evolution (Evans et al., 2016). Other studies have shown that genetic variation in *Populus*, often within a single population, influences arthropod communities (Busby et al., 2015; Dickson & Whitham, 1996; Keith et al., 2010; Shuster, Lonsdorf, Wimp, Bailey, & Whitham, 2006; Wimp et al., 2007), trophic interactions (Bailey et al., 2006), interaction networks (Lau, Keith, Borrett, Shuster, & Whitham, 2016), interactions among communities such as arthropods and endophytes (Lamit et al., 2015), ecosystem processes (Schweitzer et al., 2008), and selection on herbivores (Smith et al., 2011). However, relatively little research has focused on how the tree may influence evolutionary patterns in the dependent community (Evans et al., 2008; Smith et al., 2011).

For multiple reasons, studying *Chaitophorus populicola* on cottonwoods provides an excellent opportunity to examine how host‐tree genetics may influence local adaptation and rapid evolution in a dependent community member. First, during the summer, *C. populicola* are relatively sessile, making them easy to manipulate and track. Further, they are cyclically parthenogenic, reproducing asexually in the summer months. Consequently, during this time, it is relatively easy to monitor the abundance of individual aphid genotypes. Further, a previous study showed that population growth of *C. populicola* is affected by interspecific variation in plant quality, with aphid growth being greatest on narrowleaf (*P. angustifolia*) and least on Fremont (*P. fremontii*) cottonwoods (Wimp & Whitham, 2001). However, the effects of intraspecific *Populus* genetic variation on *C. populicola* performance are not known.

### 2.2 Local adaptation experiment at the tree population level

To conduct the local adaptation experiment, we used a reciprocal transplant experiment in the greenhouse, using trees and aphids collected from three sites along an elevation/climate gradient. In January 2007, we took *P. angustifolia* cuttings from adult trees growing along the Weber River, UT, in 3 sites across ~90 km and 620 m of elevation. The three sites were (a) near Uintah, Utah, in the Salt Lake Valley on the west side of the Wasatch Mountains (1,380 m elevation), (b) near Morgan, Utah, on the east side of the Wasatch Mountains (1,545 m elevation), and (c) also east of the Wasatch Mountains and at the western end of the Uinta Mountains, 11 km below the Smith–Morehouse reservoir (2,000 m elevation). Previous studies have shown genetic and phenotypic variation in *P. angustifolia* among these three sites (Martinsen et al., 2001), as well as adaptation to local soils (Smith et al., 2012). Cuttings were taken to the greenhouse, placed in pots, and allowed to sprout in greenhouse conditions (~20–25°C and ambient daylight lengths). In July of the same year, 51 potted trees (15, 15, and 21 trees from the low, mid‐, and high elevations, respectively), each about 0.3 m tall, with approximately 10 leaves, were taken back to the field, to the same three sites where tree collections were made. At these sites, we located wild aphid colonies. Aphids were collected from trees within 200 m of the trees from which the cuttings were taken the previous winter. We did not collect aphids from the same trees as the cuttings for practical reasons. Specifically, the trees from which we collected cuttings were large, adult trees. Often, the lowest branches were 6 m from the ground. Hence, we searched smaller trees in the same area from where we took cuttings. Small branches containing aphids were removed from wild trees and aphids were immediately transferred onto the potted trees. Each potted tree received a single adult aphid. We separated potted trees by at least 30 cm and branches were not touching to prevent aphids from moving among trees. Because aphids in the wild are usually tended and protected by ants, individual trees were enclosed in mesh netting and returned to the greenhouse to prevent aphids from being attacked by predators. Potted trees were watered three times a week. After 30 days, the number of aphids was counted on each tree. If only one aphid was counted, it was presumed that the aphid did not reproduce in the 30‐day time period. To measure the total number of offspring, we counted the total number of aphids minus one (to account for the single aphid that was initially placed on the tree).

We used three statistical models to look for the three patterns of local adaptation as described by Kawecki and Ebert (2004). Importantly, in addition to having information on the origin of the tree and aphid populations, we created a new variable (“pairing”) for the analysis. Specifically, we categorized each aphid population—tree population pairing as either “home” or “foreign” (Figure 1). In our first analysis, we used the glm function in the lme4 package of R (Bates, Maechler, Bolker, & Walker, 2015; R Core Team, 2017) to conduct a generalized linear model (glm), modeling the number of aphid offspring (the dependent variable) with a poisson distribution. We included aphid population, pairing (categorized as home or foreign), and their interaction as independent variables. We then used the ANOVA function on this model, to determine the significance of the independent variables (Fox & Weisberg, 2019). We also used the glht function in the multcomp package of R (Hothorn, Bretz, & Westfall, 2008) to make pairwise comparisons of aphid offspring numbers between home and foreign pairings for each aphid population. If local adaptation occurred, we expected two results from these analyses. First, we expected a significant pairing effect, which would indicate a difference in aphid reproduction between home and foreign aphids. Second, in examining each aphid population individually, we expected that each aphid population would produce more offspring when transplanted onto their home versus foreign tree populations.

Our second model was very similar to the first, except we included tree population (instead of aphid population), pairing, and their interaction as independent variables. Like the first model, we conducted post hoc pairwise comparisons. If local adaptation is occurring, we expect the same statistical results as described above for the first model.

Finally, in our third model, we included aphid population, tree population, and pairing as the independent variables. This model controls for the effects of aphid and tree populations, while comparing the average reproduction of all home aphids (i.e., aphids in sympatry with their tree hosts), to the average reproduction of all foreign aphids (i.e., aphids in allopatry with their tree host; Blanquart et al., 2013). If local adaption occurred, we expected home (sympatric) aphids to produce more offspring than their foreign (allopatric) counterparts.

### 2.3 Rapid evolution experiment at the individual tree genotype level

The experiment to test for rapid evolution was conducted in a 15‐year‐old cottonwood common garden in Ogden, Utah. Cottonwood trees were originally cloned from haphazardly selected *P. fremontii*, *P. angustifolia,* and their naturally occurring hybrids in natural stands along the Weber River near Ogden, UT, and were planted in the garden in a random design. The genetic identity for all trees in the common garden was determined by RFLP analyses (Martinsen et al., 2001). We randomly selected five pure narrowleaf cottonwood (*P. angustifolia*) genotypes (1000, 1020, 1008, HE10, and WC5) for this experiment. Previous studies have shown that these genotypes have unique influences on arthropod community composition (Keith et al., 2010), interactions between aphids and their avian predators (Bailey et al., 2006; Smith et al., 2011) and aphid fitness (Smith et al., 2011). Further, these genotypes come from the same river system and similar elevation gradient as the trees used in the local adaptation experiment described above.

In late May 2005, we collected 10 aphid genotypes from *P. angustifolia* trees along a ~35 km east–west transect of the Weber River. This collection protocol was used for two reasons. First, we collected aphids early in the season (late May) to obtain individuals that had passed through a few generations of parthenogenetic reproduction as possible, thereby maximizing the population genetic variation among aphid genotypes (Via & Shaw, 1996; Vorburger, 2006). Second, we sought to increase the probability that the aphid genotypes were genetically distinct from one another by collecting from widely separated populations (Tack & Roslin, 2010).

In the common garden, we established 10 isofemale aphid lines by placing a single aphid from each aphid genotype onto separate branches of a single narrowleaf tree. The single female was allowed to reproduce asexually (i.e., produce clonal “stock colonies”) in cages (15 × 30 × 15 cm) covered with fine mesh. Aphid genotypes grew on the stock tree for 30–31 days, nearly three aphid generations. All aphid populations were maintained on the same individual tree to control for possible conditioning effects of the host plant (Karban, 1989). The genotype of the stock tree was different from those used in the experiment. The growing conditions for all the aphid genotypes were as identical as possible. Because light intensity can influence *C. populicola* growth (G. Wimp, personal communication), we placed aphid colonies on branches that standardized their growing conditions.

From the 10 isofemale genotypic lines, we selected four aphid genotypes for the experiment based on two practical criteria. First, to maximize the probability of high genetic variation among aphid genotypes, we chose aphid genotypes that represented aphid populations at both extremes and the middle of their distribution along the Weber River. Second, given the complexity of our experimental design, four aphid genotypes were the maximum number of genotypes that could be practically handled. Aphid genotypes from North Uintah (1,364 m elevation) and Site 9 (1,376 m elevation) originated from stands of mostly pure narrowleaf within the hybrid zone and were located within ~2 km of each other. The Red Barn (1,478 m elevation) genotype was located ~11 km east from the North Uintah Site, on the opposite side of the Wasatch Mountains in the pure narrowleaf zone. The Taggart (1,564 m elevation) genotype, also located in the pure narrowleaf zone, was ~36 km east of the North Uintah site.

We employed an experimental design in which individuals of four aphid genotypes were placed on replicate clones of five cottonwood genotypes. In the common garden, we selected four individual trees (clonal replicates) from each of the five cottonwood genotypes (20 total trees) that had been randomly planted in the common garden. On each replicate tree, we selected four branches originating at nearly the same insertion point. In early July, we placed a pair of similarly aged adult aphids from each of our four selected aphid genotypes onto a branch (1 aphid genotype per branch) such that a single replicate tree supported four distinct aphid genotypes (*N* = 80). Therefore, the initial genotype composition on each tree was identical (i.e., each tree had the same number and frequency of the four different aphid genotypes). Aphids were enclosed in mesh bags (15 × 30 × 15 cm) on their branch to prevent predation or mixing of aphid genotypes and allow for reliable, accurate counting of individuals from each aphid genotype. We censused aphid abundance, for every aphid genotype and on ever tree, daily over a period of 17 days and then every two days until 21 days had elapsed, for a total of 19 censuses. We combined the abundances of all four aphid genotypes to measure aphid genotype composition on each tree.

We analyzed changes to aphid genotype composition in part by using nonmetric multidimensional scaling (NMDS; Clarke, 1993; Faith, Minchin, & Belbin, 1987; Minchin, 1987). Several studies have used NMDS to help analyze variation in community composition, that is, the combined abundances or frequencies of different species in a community (Barbour, Baker, O'Reilly‐Wapstra, Harvest, & Potts, 2009; Busby et al., 2015; Keith et al., 2010). Compared with other multivariate techniques, for example, canonical discriminant analysis and principal component analysis, NMDS is robust and less likely to create spurious sources of variation (Shuster et al., 2006). Here, we use NMDS to help analyze variation in the combined abundances of different aphid genotypes. In the context of this experiment, NMDS incorporates the abundances of all four aphid genotypes and summarizes it into a single NMDS score for each census date x tree combination. In other words, a single NMDS score represents the aphid genotype composition on a single tree, at a single census date. Importantly, NMDS scores that are close together represent aphid genotype compositions that are relatively similar, and conversely, NMDS scores that are far apart represent aphid genotype compositions that are relatively different. Here, because they started with the same aphid genotype composition, each tree had the same NMDS score at the beginning of the experiment (day 0).

Using the NMDS score as the dependent variable, we performed repeated measures ANOVA (JMP 5.0.1, SAS Institute, Cary, NC) to determine the influence of tree genotype on changes to aphid genotype composition (i.e., aphid evolution). Tree genotype (whole plot), time (subplot), and tree genotype × time interaction were the treatment factors. Tree (the replicate) nested within tree genotype (the whole‐plot error term) was included as a random variable to provide the appropriate degrees of freedom for the whole‐plot factor, tree genotype (Gotelli & Ellison, 2004). A significant interaction effect would provide evidence that the change in aphid genotype composition through time (i.e., aphid evolution) differs across tree genotypes. Lastly, to help test the hypothesis that aphid evolution would differ among tree genotypes, we used a Tukey's test to compare the NMDS scores (aphid genotype composition) among tree genotypes on the last day of the study.

## 3 RESULTS

### 3.1 Evidence for local adaptation

**FIGURE 2:**
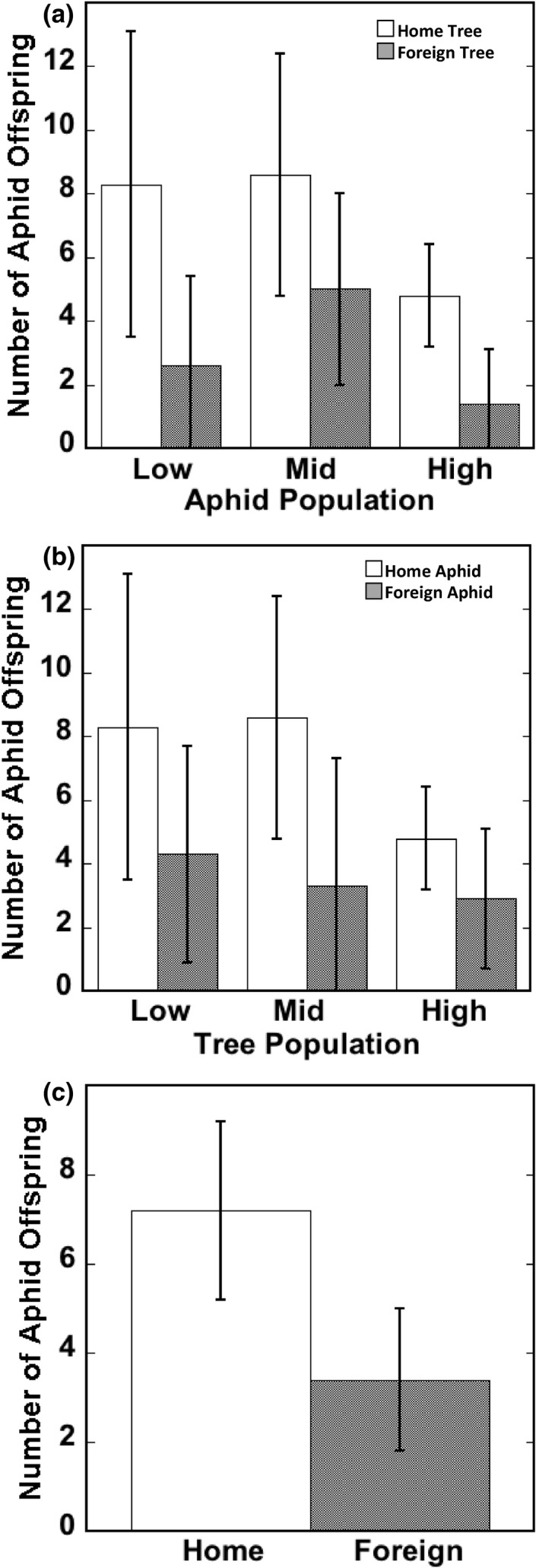
Results from the three approaches used to detect local adaptation. (a) It shows the mean number of aphid offspring (± 95% confidence interval) for each of the three aphid populations transplanted onto their home and foreign tree populations. Pairwise comparisons at the low, mid‐, and high elevations resulted in *p*‐values of <.0001, .0212, and .0105, respectively. (b) It shows the mean number of aphid offspring (± 95% confidence interval) produced on each tree population when aphids were native (home) or non‐native (foreign) for that tree population. Pairwise comparisons at the low, mid‐, and high elevations resulted in *p*‐values of .0430, <.0001, and .2470, respectively. (c) It shows the mean number of aphid offspring (± 95% confidence interval) for all home aphid treatments compared with all foreign aphid treatments

In support of our hypothesis, we found three lines of evidence that aphid populations are locally adapted to their home tree populations. First, when each aphid population was transplanted onto home and foreign tree populations, (i.e., aphid–tree pairings among different tree populations) we found that, on average, aphids produced more offspring when transplanted onto home trees ($X_{1}^{2}$ = 37.13, *p* < .0001). Results from the *post hoc* pairwise comparisons show that for all three aphid populations, aphids transplanted onto home trees produced 1.7–3.4x as many offspring as aphids transplanted onto foreign trees (Figure 2a).

Second, when comparing aphid reproduction of home and foreign aphids growing on the same tree (i.e., aphid–tree pairings within single tree populations), we also found evidence of local adaptation (pairing effect: $X_{1}^{2}$ = 29.29, *p* < .0001; Figure 2b). *Post hoc* pairwise comparisons revealed that in the low, mid‐, and high elevation trees, home aphids produced 1.9, 2.6, and 1.7x more offspring, respectively, than foreign aphids transplanted onto the same tree population (Figure 2b).

Third, when lumping data from all three populations together, home (sympatric) aphids produced more than 2x as many offspring than foreign (allopatric) aphids ($X_{1}^{2}$ = 31.82, *p* < .0001; Figure 2c).

### 3.2 Evidence for rapid aphid evolution driven by tree genotype

**FIGURE 3:**
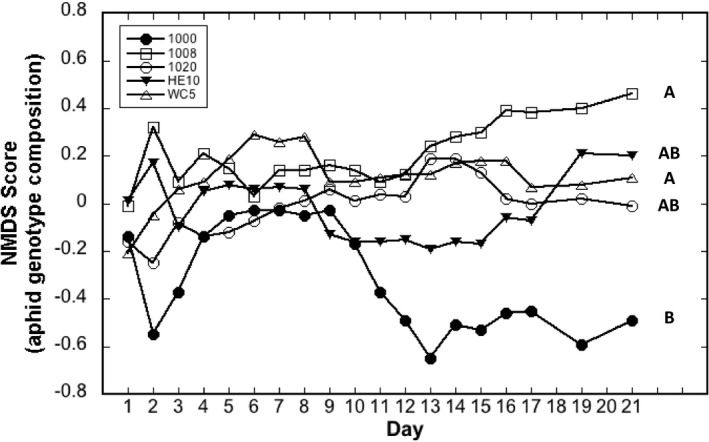
Plot of aphid genotypic composition, as derived from the NMDS analysis, across sampling dates. Each line represents the aphid genotype composition on one tree genotype. Lines that are separated represent aphid genotype compositions that are relatively different. Lines that do not share a common letter indicate significant differences in aphid genotype composition at the end of the study (Tukey's HSD, *p* < .05)

In support of our hypothesis of rapid aphid evolution, aphid genotype composition changed across time (i.e., aphids rapidly evolved) and aphid evolutionary trajectories differed across individual tree genotypes. We found a tree genotype x time interaction (*F*_72,264.8_ = 1.40, *p* = .0310) when we used NMDS score (i.e., the composition of aphid genotypes) as a response variable in a mixed model ANOVA, indicating that the change in aphid genotype composition across time (i.e., aphid evolution) differed across tree genotypes. The NMDS score represents the aphid genotype composition on each tree on each census date. Aphid genotype composition was the same on all tree genotypes at the beginning of census, then diverged over time (Figure 3, Table 1). By the end of the study, tree genotypes 1008 and WC5 had different aphid genotype compositions compared with tree genotype 1000. In contrast, on tree genotypes 1020 and HE10 aphid populations had relatively similar genotype compositions and were not different from the other trees. The change in aphid population genotype composition in response to tree genotype (Figure 3, Table 1) supports our hypothesis that individual host‐tree genotypes differentially influence rapid aphid evolution.

**TABLE 1:** NMDS scores representing the aphid genotype composition

	1000	1008	1020	HE10	WC5
Sampling day					
1	−0.14 (0.19)	−0.01 (0.33)	−0.16 (0.41)	0.01 (0.31)	−0.21 (0.24)
2	−0.56 (0.24)	0.32 (0.26)	−0.25 (0.38)	0.17 (0.19)	−0.05 (0.42)
3	−0.37 (0.52)	0.09 (0.36)	−0.08 (0.31)	−0.10 (0.47)	0.06 (0.41)
4	−0.14 (0.47)	0.21 (0.17)	−0.14 (0.26)	0.05 (0.13)	0.09 (0.27)
5	−0.05 (0.11)	0.15 (0.11)	−0.12 (0.21)	0.08 (0.07)	0.19 (0.24)
6	0.03 (0.11)	0.03 (0.09)	−0.08 (0.15)	0.06 (0.10)	0.29 (0.18)
7	−0.03 (0.10)	0.14 (0.21)	−0.02 (0.15)	0.07 (0.12)	0.26 (0.11)
8	−0.05 (0.10)	0.14 (0.29)	0.01 (0.13)	0.06 (0.10)	0.28 (0.18)
9	−0.03 (0.11)	0.16 (0.21)	0.06 (0.09)	−0.14 (0.43)	0.09 (0.09)
10	−0.17 (0.36)	0.14 (0.20)	0.01 (0.12)	−0.17 (0.51)	0.09 (0.09)
11	−0.35 (0.25)	0.09 (0.12)	0.04 (0.11)	−0.16 (0.53)	0.11 (0.11)
12	−0.49 (0.44)	0.12 (0.13)	0.03 (0.13)	−0.15 (0.52)	0.13 (0.09)
13	−0.65 (0.45)	0.24 (0.19)	0.19 (0.18)	−0.19 (0.53)	0.12 (0.09)
14	−0.51 (0.35)	0.28 (0.19)	0.19 (0.19)	−0.16 (0.54)	0.17 (0.18)
15	−0.53 (0.37)	0.30 (0.20)	0.13 (0.07)	−0.17 (0.60)	0.18 (0.16)
16	−0.46 (0.42)	0.39 (0.32)	0.02 (0.20)	−0.06 (0.69)	0.18 (0.15)
17	−0.45 (0.43)	0.39 (0.31)	0.00 (0.20)	−0.08 (0.70)	0.07 (0.26)
19	−0.59 (0.40)	0.40 (0.32)	0.02 (0.24)	0.21 (0.47)	0.08 (0.27)
21	−0.49 (0.37)	0.46 (0.31)	−0.01 (0.19)	0.20 (0.44)	0.11 (0.32)

Note

This shows the mean NMDS scores, with the 95% confidence interval in parentheses, for aphids on each tree genotype (along the top) for each sampling day (along the left side).

## 4 DISCUSSION

Two important results emerged from this study. First, in the greenhouse, aphids performed best on their home tree populations, providing strong evidence for local adaptation in *C. populicola* to their host‐tree population (Figure 2). Because the tree populations used in this experiment have differentiated along a climate gradient (Evans et al., 2016; Martinsen et al., 2001), our results suggest that plants evolving in response to climate will cause the plants' associated arthropods to evolve as well. Second, in a common garden, host‐tree genotype influenced differential aphid reproduction (i.e., natural selection) and evolution in just two generations, indicating that individual host‐tree genotypes can drive rapid aphid evolution (Figure 3, Table 1). If host‐mediated rapid evolution of aphids is observed over short timespans in an experimental common garden, it is also likely to occur in nature.

### 4.1 Rapid evolution to individual tree genotypes

Our results are consistent with the hypothesis of rapid aphid evolution (Thompson, 1998) as a result of genetic variation in individual host‐plant genotypes. Specifically, we found that host‐plant genotype significantly altered aphid genotypic composition (i.e., aphid evolution) after just two aphid generations (21 days, Figure 3, Table 1). The rapidity of the change in aphid genotype composition is testament to the strength of natural selection imposed by tree genotype. Further, we found unique evolutionary trajectories of aphids on different tree genotypes, which suggests that tree genotypes have unique selection pressures on aphid populations that result in divergent selection on aphids. Importantly, these patterns were driven largely by a single tree genotype, which created the most unique aphid evolutionary trajectory. Aphid evolution diverged the most between two tree genotypes (1000 and 1008; Figure 3). Interestingly, these two tree genotypes originated from the same site and in the wild grow about 50 m from each other. Future studies should include larger number of host‐plant genotypes to help determine the prevalence of unique evolutionary trajectories in the plant's associated community members. That said, these results show that tree genetic variation within the same site is sufficient to cause unique evolutionary trajectories of the tree's dependent arthropod community members. This new work suggests that heritable insect communities on cottonwood genotypes (Whitham et al., 2012) may be a consequence not only of differential establishment on those genotypes, but also of differential evolution on those genotypes. These patterns show that intraspecific genetic variation in the host plant can add to the variation in natural selection and evolution across space and time (Thompson, 2005).

### 4.2 Previous studies

Our results confirm those of other studies demonstrating that host‐plant genetic variation has a strong influence on herbivore population dynamics and performance (Donaldson & Lindroth, 2007; Evans et al., 2008; Garrido et al., 2012; Laukkanen et al., 2012; Ryan, Emiljanowicz, Härri, & Newman, 2014; Service, 1984; Smith et al., 2011; Stireman, Nason, & Heard, 2005; Underwood & Rausher, 2000; Via, 1990). Results of studies designed to identify local adaptation of herbivores on their host plants have been mixed (Garrido et al., 2012; Karban, 1989; Laukkanen et al., 2012, 2018; Strauss, 1997). For example, Strauss (1997) studied a relatively mobile insect herbivore and did not find evidence for local adaptation. That study used plant and herbivore genotypes collected across an 8.6 km gradient; the relatively high mobility of the herbivore may have increased gene flow among insect demes and diluted local adaptation. Karban (1989), on the other hand, found that thrips were locally adapted to a particular clone of their host plant. Interestingly, the different plant clones used in the experiment originated within 500 m of each other, showing that sufficient plant genetic variation exists in a relatively small area to have evolutionary implications for other species. Using similar methods to ours, Laukkanen et al. (2012) performed a reciprocal transplant experiment of three populations of both plants and herbivores, separated by as much as 50 km. They found evidence of local adaption of a specialist herbivore to different populations of its host plant.

Our results are unique from previous studies for at least three reasons. First, the plant populations used in this study are known to be genetically differentiated along a climate gradient (Evans et al., 2016; see Grady et al., 2011 for similar findings with *P. fremontii*). Thus, our results show that plant evolution in response to climate variation can have evolutionary implications for the plant's dependent community. More specifically, our results show that one species evolving in the wake of climate change could have a ripple effect to cause coincident evolution in associated species. Second, we showed how rapidly herbivores can evolve in response to a plant genetic gradient. By monitoring genotype frequencies every day, we found evidence for evolution in just 21 days (~2 generations). While multiple studies have detected rapid evolution by measuring trait frequencies before and after a selection event (Franks, Sim, & Weis, 2007; Smith et al., 2015; Sthultz, Gehring, & Whitham, 2009), it is much less common to monitor genotype frequencies on regular, small time intervals to obtain precise estimates of the pace of evolution. Finally, cottonwoods (*Populus* spp.) have become a model system to investigate how intraspecific genetic variation can foster variation in the surrounding ecosystem. Dozens of studies have shown how genetic variation in cottonwoods can have major influence on the surrounding ecological patterns, such as community structure and stability (Compson et al., 2016; Keith et al., 2010; Schweitzer et al., 2008), species interactions (Bailey et al., 2006; Smith et al., 2011), and ecosystem functions (LeRoy, Whitham, Wooley, & Marks, 2007; Lojewski et al., 2012). This is one of the first studies (Smith et al., 2011) to show that intraspecific variation in cottonwoods can influence the evolutionary dynamics of the dependent community.

### 4.3 Implications

Results from this research have important implications for understanding ecological and evolutionary consequences of global change. Other studies have shown that plant genetic structure of both short‐ and long‐lived species can rapidly change in the wake of human disturbance, such as through introduced species (Smith et al., 2015) and climate change (Franks et al., 2007; Sthultz et al., 2009). If herbivore evolution is linked to plant genetics, as shown in this study, rapid plant evolution will likely have cascading effects to cause evolution of the plants' dependent community (Abrahamson, Blair, Eubanks, & Morehead, 2003; Evans et al., 2008). Based on these and similar results, we suggest several avenues of future research. We suggest that future studies measure the rate of *adaptive* evolution in response to global change. The rate of evolution has been shown in some contexts, such as plants (Franks et al., 2007; Smith et al., 2015; Sthultz et al., 2009) and animals (Lopes, Sucena, Santos, & Magalhães, 2008), but more studies are needed to better understand the frequency and pace of adaptive evolution.

In addition, we recommend that future studies examine how plant evolution will affect the plant's associated community. Resurrection studies could be used for this purpose (Bustos‐Segura, Fornoni, & Núñez‐Farfán, 2014). Resurrection studies grow plants from seeds collected from current and previous time periods (the “resurrected” plants). The two populations of plants are then compared to examine how plants have evolved through time (Bustos‐Segura et al., 2014; Franks, Hamann, & Weis, 2018; Franks et al., 2007). In addition to measuring plant traits and plant evolution, these studies could examine how ecological and evolutionary dynamics of arthropods differ in the present and resurrected lines of plants. It is important to note that the effect of plants on arthropods may be direct and/or indirect. For example, changes in herbivore population size and/or genetic variation as a result of plant evolution may cascade to influence other community members (Hazell & Fellowes, 2009; Hufbauer & Via, 1999; Keith et al., 2017; Smith et al., 2011; Stireman et al., 2005). In one study, plant genetics influenced the presence of the herbivore *C. populicola,* leading to an increase in tending ants whose presence shaped a much larger arthropod community (Wimp & Whitham, 2001). In terms of genetics, Hazell and Fellowes (2009) showed that variation among pea aphid genotypes not only influenced predator success, but also altered predator community composition. Thus, changes in herbivore genotype composition (i.e., herbivore evolution) could cause further changes to other aspects of the local community.

Finally, we suggest expanding the genetic similarity rule (Bangert et al., 2006) to evolution. Bangert et al. (2006) posited that plants with similar genetics will support similar communities. In short, functional plant traits often influence the occurrence and abundance of other community members. Thus, plants with similar genetically based traits will support similar communities, a pattern that has been documented in cottonwood and other systems (e.g., Barbour et al., 2009; Zytynska, Fay, Penney, & Preziosi, 2011). Although most of these studies have used neutral genetic markers to characterize genetic similarity, Barbour et al. (2009) showed that with quantitative traits of phytochemistry and foliage morphology of *Eucalyptus globulus*, genetic similarity was a much stronger predictor of community similarity. As an extension, because plant traits can act as agents of natural selection on associated species, we expect plants with similar genetically based traits to support similar species interactions and evolutionary trajectories in the interacting species. Among other insights, studies such as these will improve understanding of eco‐evolutionary dynamics in the wake of global change.

## CONFLICT OF INTEREST

Authors do not have any conflicts of interests.

## AUTHOR CONTRIBUTIONS

**Stuart C. Wooley:** Conceptualization (equal); data curation (equal); formal analysis (equal); investigation (equal); methodology (equal); project administration (equal); supervision (equal); writing – original draft (equal); writing – review & editing (equal). **David Solance Smith:** Conceptualization (equal); data curation (equal); formal analysis (equal); funding acquisition (equal); investigation (equal); project administration (equal); writing – original draft (equal); writing – review & editing (equal). **Eric V. Lonsdorf:** Formal analysis (equal); writing – original draft (supporting). **Sarah C. Brown:** Data curation (supporting); investigation (equal); methodology (supporting); project administration (equal). **Thomas G. Whitham:** Conceptualization (supporting); funding acquisition (equal); writing – review & editing (equal). **Stephen M. Shuster:** Formal analysis (supporting); funding acquisition (equal); writing – review & editing (equal). **Richard L. Lindroth:** Conceptualization (equal); funding acquisition (lead); methodology (equal); writing – review & editing (equal).

## Data Availability

Data are available on Dryad https://doi.org/10.5061/dryad.18931zcv2.
